# Establishing trust in artificial intelligence-driven autonomous healthcare systems: an expert-guided framework

**DOI:** 10.3389/fdgth.2024.1474692

**Published:** 2024-11-27

**Authors:** Turki Alelyani

**Affiliations:** Department of Information Systems, College of Computer Science and Information Systems, Najran University, Najran, Saudi Arabia

**Keywords:** autonomous systems, artificial intelligence, framework, trustworthiness, healthcare

## Abstract

The increasing prevalence of Autonomous Systems (AS) powered by Artificial Intelligence (AI) in society and their expanding role in ensuring safety necessitate the assessment of their trustworthiness. The verification and development community faces the challenge of evaluating the trustworthiness of AI-powered AS in a comprehensive and objective manner. To address this challenge, this study conducts a semi-structured interview with experts to gather their insights and perspectives on the trustworthiness of AI-powered autonomous systems in healthcare. By integrating the expert insights, a comprehensive framework is proposed for assessing the trustworthiness of AI-powered autonomous systems in the domain of healthcare. This framework is designed to contribute to the advancement of trustworthiness assessment practices in the field of AI and autonomous systems, fostering greater confidence in their deployment in healthcare settings.

## 1 Introduction

The rapid advancement and integration of Autonomous Systems (AS) in various domains have sparked significant interest in assessing their trustworthiness. AS are implemented using Artificial Intelligence (AI) as a key mechanism, either fully autonomous or partially autonomous. AI enables AS to make intelligent decisions, learn from data and adapt to changing environments without direct human intervention (1). The term “autonomous system” refers to a system that can make decisions without human involvement (2). We can think of the ability to make autonomous decisions as a range of possibilities. On one end of this range, we have highly autonomous systems that handle the decision-making process and the execution of resulting actions. On the other end, there are advisory systems where the decision-making process is partially or mostly delegated to the system, but the human in control remains responsible for carrying out the recommended actions (3). In fully autonomous systems, AI operates independently and performs tasks without human control or intervention. These systems can analyze complex data, perceive their environment, and make informed decisions to accomplish their objectives. On the other hand, partly autonomous systems combine AI capabilities with human control, where humans and AI collaborate to achieve specific goals (2, 3). AI assists humans by providing recommendations, automating routine tasks, or enhancing decision-making processes in such systems. AS has become more prevalent and assumes greater responsibilities for our safety, so it is essential to develop robust methods for evaluating their reliability, and ethical behavior. The verification and development community faces the challenge of ensuring the trustworthiness of AS in a comprehensive and objective manner (4). Appropriate standards and metrics are required to evaluate trustworthiness across a wide range of applications to address this challenge. In recent years, research efforts have focused on defining the concept of trustworthiness and establishing frameworks to assess it. The concept encompasses multiple dimensions: safety, security, privacy, transparency, and fairness (2, 4). In the context of autonomous systems, trustworthiness extends beyond functional correctness and encompasses the system’s ability to make informed and ethical decisions, adapt to changing conditions, and operate within legal and societal boundaries (5, 6). Standards and frameworks play a vital role in guiding the development and assessment of AS trustworthiness (7, 8). Organizations such as ISO, IEEE, and NIST have been actively involved in establishing guidelines and standards to ensure the safety and reliability of AS in various domains (9). For instance, ISO 21448, also known as the Safety of the Intended Functionality (SOTIF) standard, focuses on addressing hazards related to AS’s intended operational design domain (9). These standards provide a foundation for evaluating and improving trustworthiness. While progress has been made in developing standards and frameworks, challenges remain in their practical implementation. One challenge lies in adapting general trustworthiness concepts to specific domains, such as healthcare (10). Autonomous systems in healthcare requires specialized considerations to ensure patient safety and the ethical use of data (11). It is essential to gather insights from experts in the field to address the gap between general frameworks and domain-specific requirements. Expert interviews provide valuable perspectives on the specific challenges and considerations related to trustworthiness in autonomous systems for healthcare systems. This study aims to develop a comprehensive framework that contributes to developing guidelines and practices that enhance AI-based Autonomous systems’s reliability, safety, and ethical behavior in healthcare.

## 2 Background

### 2.1 Artificial intelligence-based autonomous systems

Artificial Intelligence based Autonomous Systems (AS) are sophisticated systems designed to autonomously learn, adapt, and make decisions within predefined boundaries. These systems have the potential to transform healthcare delivery by enhancing precision, streamlining processes, and delivering personalized care to patients. Yet, guaranteeing their safety and dependability is paramount in fostering trust in these technologies. Previous studies have outlined various essential factors that need to be integrated into the development of such systems:

• Data Quality and Bias: Flawed or biased data can lead to inaccurate AI outputs and potentially harmful recommendations (12). Therefore, it is essential to carefully curate and validate the data used to train AI models to ensure that they are representative and free from bias.
• Algorithmic Transparency and Models Explainability: Clinicians need to understand the reasoning behind AI decisions to maintain trust and oversight in the system (13). Transparent algorithms and explainable models enable clinicians to interpret and verify the AI’s recommendations, enhancing their confidence in using AI-based AS for patient care.
• Robustness and Security: AI systems need to be resilient against errors, cyberattacks, and unexpected situations to ensure patient safety (14). Robust security measures and contingency plans should be in place to mitigate risks and ensure the reliable operation of AI-based AS in healthcare settings.

AI-based AS applications in healthcare are making a significant impact, revolutionizing the field and improving patient outcomes as illustrated in these studies (12, 15, 16). One area where AI-based AS is particularly impactful is diagnostic imaging. AI algorithms can analyze medical scans such as X-rays, MRIs, and CT scans with greater accuracy and speed, helping radiologists detect and diagnose diseases earlier and more accurately than ever before. For example, AI-powered systems can flag areas of concern in an X-ray, highlighting potential fractures or abnormalities that may require further investigation. This speeds up the diagnostic process and reduces the likelihood of human error, leading to more reliable diagnoses. Another area where AI is transforming healthcare is in drug discovery. The process of developing new medications is traditionally slow and costly, requiring extensive research and testing. AI is changing this by analyzing vast biological information and chemical compounds datasets to identify potential drug candidates much faster. By rapidly screening and simulating the interactions between drugs and biological targets, AI can significantly accelerate the drug discovery process, potentially leading to the development of life saving medications in a fraction of the time it would take using traditional methods (14). Robot-assisted surgery is another area where AI-based AS is making a significant impact in healthcare. Surgical robots, which are controlled by surgeons but equipped with AI features like tremor filtering and motion scaling, are revolutionizing surgical procedures by improving precision and reducing the risk of complications. For example, in minimally invasive surgeries, where precision is crucial, AI-powered surgical robots can enhance the surgeon’s movements to ensure more accurate incisions and suturing, leading to better patient outcomes and faster recovery times (13).

### 2.2 Trustworthiness dimensions and concepts

Numerous dimensions and concepts contribute to the understanding of trustworthiness in AS. Research studies highlight the importance of safety, security, privacy, transparency, and fairness as key dimensions of trustworthiness (5). These dimensions align with the principles of responsible AI and emphasize the ethical considerations in AS development and deployment (4, 6, 17, 18). Standards and frameworks play a crucial role in guiding the assessment and improvement of trustworthiness in AS. ISO 21448, also known as the Safety of the Intended Functionality (SOTIF) standard, focuses on addressing hazards related to the intended operational design domain of AS, ensuring the system’s behavior is safe (17). The NIST Framework for Improving Critical Infrastructure Cybersecurity provides guidelines for managing and assessing cybersecurity risks in AS. Recent research discusses metrics and measures for assessing trustworthiness in AS (19–21). Trustworthiness in AS extends beyond technical aspects and encompasses ethical considerations. Research experts emphasize the importance of ethical principles in scientific cyber infrastructures, emphasizing the need for transparent decision-making and accountability (19). In healthcare, ethical considerations in algorithmic decision making become even more critical highlightings the need for fairness, accountability, and transparency (20, 22–24). Another major study addresses the ethical considerations in machine learning for clinical medicine, including privacy and fairness concerns (21).

Trustworthiness assessment and domain-context factors Assessing the trustworthiness of AS presents several challenges. In one article reviewed, authors emphasize the difficulty in establishing comprehensive and objective metrics for trustworthiness assessment (21). The complexity of AS and their interaction with human users and the environment adds to the challenge of ensuring trustworthiness (20, 25). Another study discusses the impact of smart city systems on privacy, highlighting the challenges of ensuring trustworthiness in a connected environment (26). A study published in 2,019 provides a review of trustworthiness assessment and metrics for digital systems (27). Moreover, domain-specific considerations are crucial in assessing trustworthiness. In the context of health- care, specialized requirements emerge. Prior research reviewed the importance of increasing value and reducing waste in research design and analysis, emphasizing the need for rigorous evaluation of AS in healthcare settings (28). Secondly, research highlight the need for trustworthy AI in healthcare, considering the criticality of accurate diagnoses and treatment decisions (3). Additional studies address the concept of meaningful human control over autonomous systems, which is particularly relevant in healthcare contexts (11). There are several factors that were studied in literature that contribute to the trustworthiness:

1. Safety: Refers to the assurance that the autonomous system operates without causing harm or posing risks to individuals or the environment (29).
2. Security: Involves protecting the autonomous system from unauthorized access, data breaches, and malicious activities that may compromise its functionality or integrity (6).
3. Privacy: Entails safeguarding sensitive and personal information collected, processed, or stored by the autonomous system, ensuring compliance with privacy regulations, and maintaining confidentiality (17).
4. Transparency: Refers to the ability to understand and interpret the decision-making processes, algorithms, and inner workings of the autonomous system, enabling users to comprehend how and why specific outcomes or recommendations are generated (11).
5. Fairness: Involves ensuring that the autonomous system’s decisions, actions, and outcomes are unbiased, equitable, and do not disproportionately favor or discriminate against individuals or groups based on protected characteristics (30, 31).

## 3 Materials and method

### 3.1 Materials

This study employed a qualitative research design using semi-structured interviews. First, the researcher obtained approval from the Institutional Review Board (IRB) of Najran University, under protocol code 012979-029337-DS. Informed consent was obtained from all participants prior to the interviews, ensuring confidentiality and anonymity. The research adhered to ethical guidelines to safeguard participants’ rights and well-being, as well as to ensure the responsible handling of data. Purposeful sampling was used to identify and select domain experts with extensive knowledge and experience in autonomous systems technologies in healthcare, specifically in the development, evaluation, and implementation of these systems in healthcare settings. Potential participants were contacted via LinkedIn and invited to participate in the study. Ultimately, 15 experts were recruited, and interviews were conducted with them. The sample size was determined by data saturation, where recurring themes began to emerge (35). Experts from diverse backgrounds were included, and the demographics of the final participants are presented in Table 1.

**Table 1:** Experts’ title, years of experience, country, associated industries, and race/ethnicity.

Expert	Job title	Years of experience	Industry	Country	Race/ethnicity
Expert 1	AI researcher	10	Healthcare	USA	Caucasian
Expert 2	Data scientist	8	Medical technology	USA	African American
Expert 3	Clinical oncologist	15	Healthcare	USA	Asian
Expert 4	Ethical AI consultant	12	AI ethics	Canada	Caucasian
Expert 5	Regulatory affairs specialist	10	Healthcare regulations	Canada	Middle eastern
Expert 6	Human-computer interaction researcher	7	Academia	Germany	Caucasian
Expert 7	Patient advocate	5	Non-profit	Austria	Caucasian
Expert 8	AI systems engineer	9	Medical device manufacturing	Austria	African
Expert 9	Health informatics specialist	10	Healthcare IT	India	Indian
Expert 10	Legal and compliance officer	13	Legal services	India	Indian
Expert 11	Radiologist	8	Healthcare	India	Indian
Expert 12	AI policy analyst	6	Public policy	USA	Hispanic
Expert 13	Biomedical engineer	11	Medical research	USA	Caucasian
Expert 14	Chief medical officer	20	Healthcare	USA	African American
Expert 15	AI product manager	7	AI technology	UK	Asian

The study developed the interview guide based on the measures outlined by The National Institute of Standards and Technology (NIST) (9), which encompass various aspects such as validity and reliability, safety, security and resiliency, accountability and transparency, explainability and interpretability, privacy, and fairness (Additional file Supplementary Appendix) (32). The interview questions were designed to explore various dimensions of trustworthiness, challenges, ethical considerations, and the applicability of existing standards and frameworks. The interviews were audio-recorded with participants’ consent and later transcribed for analysis.

### 3.2 Study design

Semi-structured interviews were conducted with 15 experts to gather their insights, perspectives, and evaluations of trustworthiness in autonomous systems (AS) for healthcare. The study employed quantitative thematic analysis, which allowed for the systematic identification and quantification of emerging themes from the interview transcripts. This method enabled a structured and rigorous approach to analyzing the data, ensuring that the findings were both comprehensive and replicable.

To explore themes related to the National Institute of Standards and Technology (NIST) measures, a combination of inductive and deductive approaches was used. Predefined codes, based on the research objectives and existing literature, were initially applied to the data. However, additional codes were developed during the analysis to capture new and emerging themes that were not initially anticipated. This flexible coding approach allowed the study to remain open to unexpected insights while staying aligned with the original research framework.

The use of quantitative thematic analysis not only facilitated the systematic organization of the data into themes and sub-themes but also provided a means to quantify the occurrence of specific themes, enhancing the reliability and transparency of the findings. By applying this method, the study was able to draw out key patterns related to trustworthiness in AS for healthcare while ensuring that the analysis remained objective and reproducible. This robust approach helped to strengthen the study’s conclusions by providing a detailed, yet quantifiable, understanding of expert perspectives on trustworthiness in AS for healthcare.

## 4 Results

**Figure 1:**
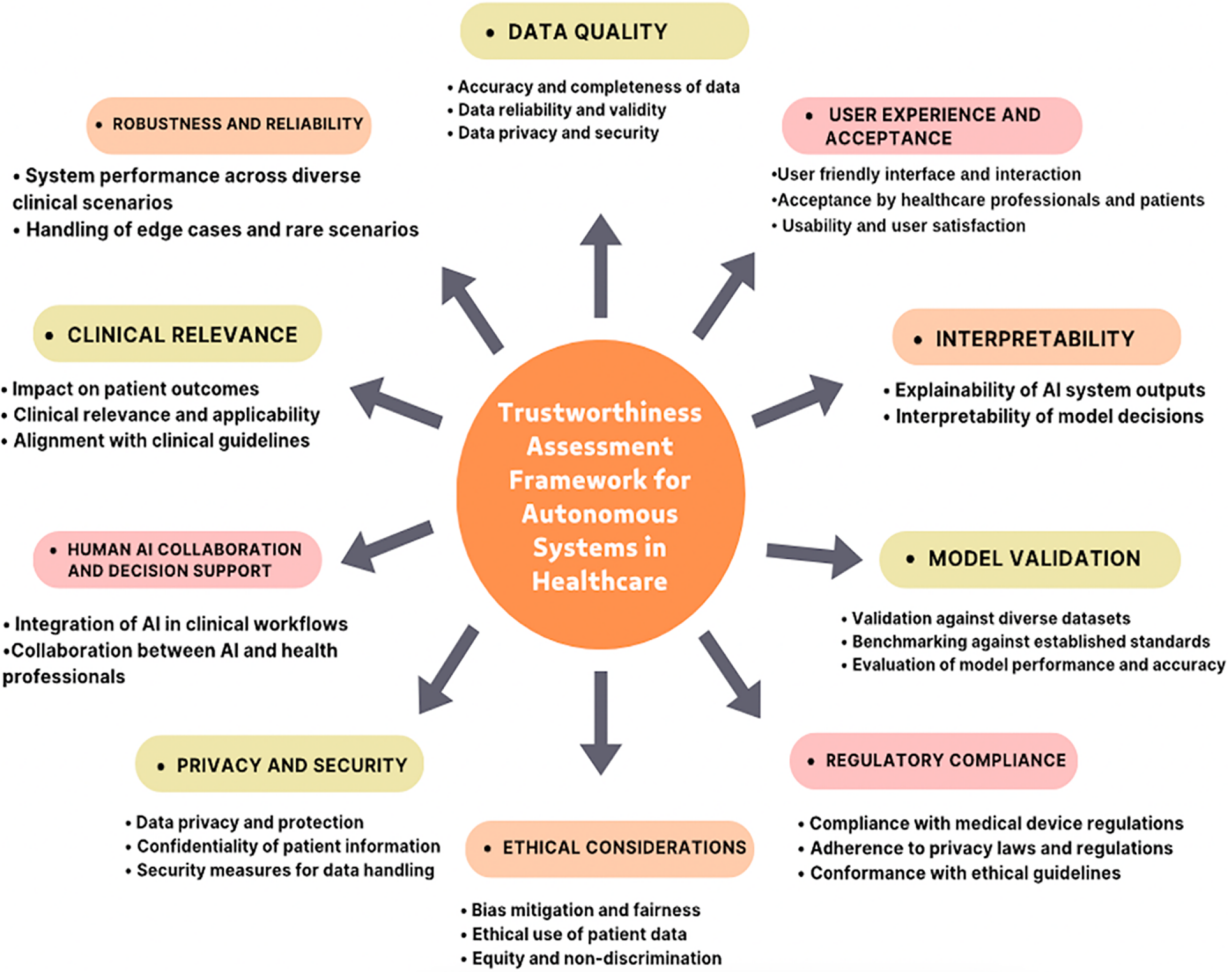
A comprehensive framework for assessing the trustworthiness of Autonomous Systems (AS) in healthcare. This framework integrates expert insights to enhance trustworthiness assessment practices, thereby fostering greater confidence in the deployment of AI-powered autonomous systems in healthcare settings.

In this study, I conducted interviews with 15 experts in the field of healthcare, technology, and autonomous systems (AS) to gather their insights, perspectives, and evaluations of trustworthiness in AI based AS for healthcare. The interviews aimed to identify critical factors that contribute to the trustworthiness of AS and refine the trustworthiness assessment framework accordingly. Based on the integrated findings, a trustworthiness assessment framework for AS in healthcare was developed. The framework encompassed the identified dimensions, factors, standards, and metrics that contribute to trustworthiness. The framework was designed to assist stakeholders in evaluating and ensuring the trustworthiness of AS in healthcare applications. Figure 1 illustrates all the themes related to the framework based on the factors extracted from experts interview.

## 5 Trustworthiness factors

Thematic analysis of the interview data revealed several key factors that emerged as critical for assessing the trustworthiness of AS in healthcare. These factors encompassed various dimensions and considerations, including data quality and integrity, model validation and performance, interpretability and explainability, clinical relevance and impact on patient outcomes, ethical considerations and fairness, privacy and security, human-AI collaboration and decision support, regulatory compliance, robustness and reliability, and user experience and acceptance.

### 5.1 Data quality and integrity

Experts highlighted the importance of data quality for trustworthiness. One expert stated, “High-quality data is crucial for the trustworthiness of AI systems in healthcare.” They emphasized the need for data to be accurate, complete, and reliable, as poor data quality can lead to erroneous conclusions and undermine trust in the system. Experts also discussed the importance of data integrity, ensuring that data is not altered or corrupted, which is essential for maintaining trust in AI systems.

### 5.2 Model validation and performance

Rigorous validation processes are essential. “Validation builds trust,” stated an expert. AI models need validation against diverse datasets and established standards for accuracy and reliability. This ensures the model generalizes well to real-world scenarios and avoids overfitting to the training data. Continuous monitoring and evaluation ensure ongoing trust by identifying performance degradation over time.

### 5.3 Interpretability and explainability

Clinicians need to understand how AI systems arrive at decisions. “Interpretability is key to trusting healthcare AI,” an expert noted. Transparent and understandable explanations are crucial for building trust among clinicians and patients. This allows them to evaluate the reasoning behind the AI’s recommendations and integrate them with their own medical expertise.

### 5.4 Clinical relevance and impact on patient outcomes

Experts discussed the significance of clinical relevance. One expert stated, “Trustworthiness relies on the AI model’s performance in accurately identifying markers and predicting outcomes.” They emphasized the importance of AI systems aligning with clinical guidelines and demonstrating real-world applicability to ensure positive impacts on patient outcomes, which is a key factor in assessing their effectiveness and trustworthiness.

### 5.5 Ethical considerations and fairness

Ethical considerations, such as addressing biases and ensuring fairness in AI-driven healthcare, were deemed essential for trustworthiness. Experts emphasized the need for ethical considerations. One expert stated, “Trustworthiness hinges on the system’s ability to provide equitable care.” They discussed the importance of addressing biases and ensuring fairness in AI-driven healthcare to build trust among patients, healthcare providers, and other stakeholders. Experts also highlighted the need for AI systems to provide unbiased and fair care, regardless of factors like race or socioeconomic status.

### 5.6 Privacy and security

Experts highlighted the importance of privacy and security measures. One expert noted, “Trustworthiness requires robust privacy and security measures.” They discussed the need for AI systems to protect patient data and maintain confidentiality, emphasizing that patients must have confidence that their data is handled securely in AI systems used in healthcare.

### 5.7 Human-AI collaboration and decision support

Experts discussed the significance of human-AI collaboration. One expert remarked, “Human-AI collaboration is essential to ensure that decisions are made based on a combination of clinical expertise and AI-driven insights.” They emphasized that AI systems should augment the capabilities of healthcare professionals rather than replace them, enabling shared decision-making and improving patient outcomes.

### 5.8 Regulatory compliance

Experts emphasized the importance of regulatory compliance. One expert noted, “Compliance with regulations is essential for trustworthiness.” They discussed the need for AI systems to adhere to relevant regulatory frameworks and guidelines, including medical device regulations and privacy laws, to ensure their safe and trustworthy deployment in healthcare settings.

### 5.9 Robustness and reliability

Experts stressed the need for reliability. One expert stated, “Reliability is a critical factor in trustworthiness.” They discussed the importance of AI systems performing consistently across diverse clinical scenarios and handling edge cases effectively to build trust among healthcare professionals and patients.

### 5.10 User experience and acceptance

Experts discussed the significance of user experience. One expert remarked, “User experience plays a significant role in trustworthiness.” They emphasized the importance of AI systems being user-friendly and accepted by healthcare professionals and patients, highlighting that user experience can influence trust in the system and its adoption in healthcare settings.

## 6 Discussion

This study presents a comprehensive framework for assessing the trustworthiness of Artificial Intelligence (AI)-based Autonomous Systems (AS) in healthcare, grounded in the insights and experiences of healthcare experts. The framework identifies ten key dimensions that influence trust in AI-based AS systems. Foremost, experts underscore the critical importance of data quality. Accurate, complete, and unbiased data form the foundation for training reliable AI models that produce trustworthy outcomes. Without high-quality data, models may generate misleading results, ultimately undermining trust in the system. Rigorous validation processes are also essential; AI models must be tested against diverse datasets and established benchmarks to ensure generalizability to real-world scenarios. Continuous monitoring is equally important, enabling the detection of performance degradation over time to uphold trust. Beyond technical factors, the framework stresses the importance of human understanding and control. Interpretable models are vital for clinicians, allowing healthcare professionals to understand AI recommendations and integrate them into their clinical expertise. Trust is also built through alignment with real-world impact–AI systems must demonstrate adherence to clinical guidelines and positive effects on patient outcomes (24, 33, 34). Ethical considerations are another key theme that emerged from the study. AI-based AS systems must be developed to ensure fairness, avoiding biases related to factors like race or socioeconomic status. In parallel, robust privacy and security safeguards are critical for protecting sensitive patient data and maintaining public trust in healthcare-related AI systems. The framework further emphasizes that AI should augment, not replace, healthcare professionals. Shared decision-making that leverages both human expertise and AI capabilities leads to better patient outcomes. Additionally, the framework highlights the importance of regulatory compliance. Adherence to medical device regulations, privacy laws, and ethical guidelines fosters trust by ensuring the safe and responsible deployment of AI in healthcare. Finally, the framework acknowledges the role of user experience and acceptance. AI systems that are user-friendly are more likely to be seamlessly integrated into clinical workflows and adopted by healthcare professionals, leading to successful implementation in patient care. By considering these critical dimensions, this framework offers a holistic approach to evaluating the trustworthiness of AI in healthcare. Developers and healthcare professionals can collaborate to build trust in AI, ultimately fostering its successful adoption in clinical practice. Future research should explore the framework’s applicability across various healthcare specialties, refining its criteria for specific domains to ensure its broader relevance. The overarching goal is to contribute to a future where trustworthy AI enhances patient care. Ensuring fairness and transparency in AI systems is crucial. By addressing biases, this framework can help develop AI systems that provide equitable care for all patients, regardless of their background. Additionally, AI systems should serve as tools to augment healthcare professionals rather than replace them. Shared decision-making between AI and clinicians is vital for achieving better patient outcomes and maintaining trust in the system.

## 7 Implications

The trustworthiness assessment framework for AI-based Autonomous Systems (AS) in healthcare developed in this study presents several important implications for the field.

First, the framework provides a structured, comprehensive approach for evaluating the trustworthiness of AS in healthcare. It addresses multiple critical dimensions, including data quality, interpretability, clinical relevance, ethical considerations, privacy and security, human-AI collaboration, regulatory compliance, robustness and reliability, and user experience and acceptance. By encompassing these aspects, the framework enables a holistic assessment of AS systems, offering significant value to developers, regulators, and healthcare professionals aiming to deploy trustworthy AI solutions.

Second, the framework emphasizes the need for ethical considerations during the development and deployment of AS. By focusing on bias mitigation, fairness, and transparency, the framework plays a pivotal role in building trust among patients, healthcare providers, and stakeholders. Integrating ethical dimensions into the development process ensure that the AS systems are fair, unbiased, and aligned with ethical principles.

Third, the framework underscores the importance of robust validation and performance evaluation. Rigorous validation processes—benchmarking against established standards and testing across diverse datasets—ensure the reliability and accuracy of AS systems. This fosters transparency, reproducibility, and accountability in AI model development and evaluation, which is crucial for trustworthiness.

Fourth, the framework highlights the significance of human-AI collaboration. Rather than replacing healthcare professionals, AS systems should complement their expertise, facilitating shared decision-making and improving patient outcomes. This positions AS as an essential decision support tool in healthcare.

Fifth, the framework addresses the critical aspect of user experience and acceptance. For effective adoption and utilization of AS systems, they must be designed with user-centered principles. The framework emphasizes the need for user-friendly interfaces, clear communication of AI outputs, and consideration of user preferences and needs, all of which influence trust and usability.

Finally, the framework provides valuable guidance for regulatory bodies and policymakers. By incorporating the outlined dimensions, regulatory frameworks can be developed to ensure the safe and trustworthy deployment of AS in healthcare and similar domains. While these implications are specific to healthcare, many can be extended to other sectors, facilitating responsible development and deployment of AS across various fields.

Future research should explore the application of this framework in sectors such as finance, education, and transportation, assessing its adaptability and broader utility. Further studies involving larger, diverse samples of experts and stakeholders will be necessary to validate and refine the framework’s applicability in real-world settings. Additionally, pilot implementations and case studies could provide practical insights and identify areas for improvement in healthcare.

This framework has practical applications for healthcare professionals, regulatory bodies, and policymakers. It serves as a tool to evaluate the ethical and performance standards of AI-based AS, ensuring compliance and patient safety. Exploring its applicability beyond healthcare will offer broader insights into its versatility and utility across various sectors.

## 8 Limitations

While this study provides valuable insights into the development of a trustworthiness assessment framework for Autonomous Systems (AS) in healthcare, several limitations should be considered when interpreting the findings. First, the generalizability of the framework may be restricted due to its specific focus on healthcare. While the framework addresses trustworthiness within this domain, its applicability to other sectors, such as finance, education, or transportation, may vary. Each field may present unique challenges, requirements, and ethical concerns that must be considered in the development of trustworthiness frameworks. Future studies should explore the relevance and adaptability of this framework across different industries to expand its utility. Second, the expert interviews were limited to a specific group of professionals in healthcare-related autonomous systems and technology. Although efforts were made to include experts with diverse backgrounds and experiences, the perspectives gathered may not fully represent the breadth of expertise required for a comprehensive trustworthiness assessment. Additional interviews with a broader group, including clinicians, researchers, policymakers, and patient representatives, could provide a more holistic understanding of trustworthiness in AS. Third, while the framework focuses on assessing trustworthiness, it does not provide detailed guidance on the practical implementation or operationalization of AS in healthcare. Practical aspects, such as data governance, infrastructure requirements, and regulatory compliance, are critical to the deployment of these systems but fall outside the scope of this study. Future research should address these operational concerns to enhance the framework’s real-world applicability. Finally, the qualitative nature of the expert interviews may introduce personal biases or subjective interpretations, which could influence the findings. This limitation, coupled with the relatively small sample size, impacts the generalizability of the results. Future studies should incorporate quantitative methods or larger-scale surveys to generate more robust and representative data, enabling the validation of the framework in a broader context.

## 9 Conclusion

This study addressed the critical need for a trustworthiness assessment framework for Autonomous Systems (AS) in healthcare. A framework has been developed to evaluate the trustworthiness of AS. The framework encompasses multiple “dimensions,” including data quality, interpretability, clinical relevance, ethical considerations, privacy and security, human-AI collaboration, regulatory compliance, robustness and reliability, user experience and acceptance. By considering these dimensions, the framework provides a holistic approach to assessing the trustworthiness of AS in healthcare. The results of the study also demonstrate the practical application of the “framework,” through expert interviews. Moving forward, future research should focus on refining and validating the “frame work,” expanding the sample size of experts and stakeholders, and exploring its applicability in real-world settings. Additionally, designers and policymakers should also address the potential concerns in this framework including usability, interoperability, and cost-effectiveness.

## Data Availability

The datasets presented in this study can be found in online repositories. The names of the repository/repositories and accession number(s) can be found below: https://doi.org/10.5281/zenodo.10445881.
