# Supplementary figures and images for: Establishing trust in artificial intelligence-driven autonomous healthcare systems: an expert-guided framework
Source: Front Digit Health. 2024 Nov 27;6:1474692. doi: 10.3389/fdgth.2024.1474692 (PMC11631875; doi:10.3389/fdgth.2024.1474692)

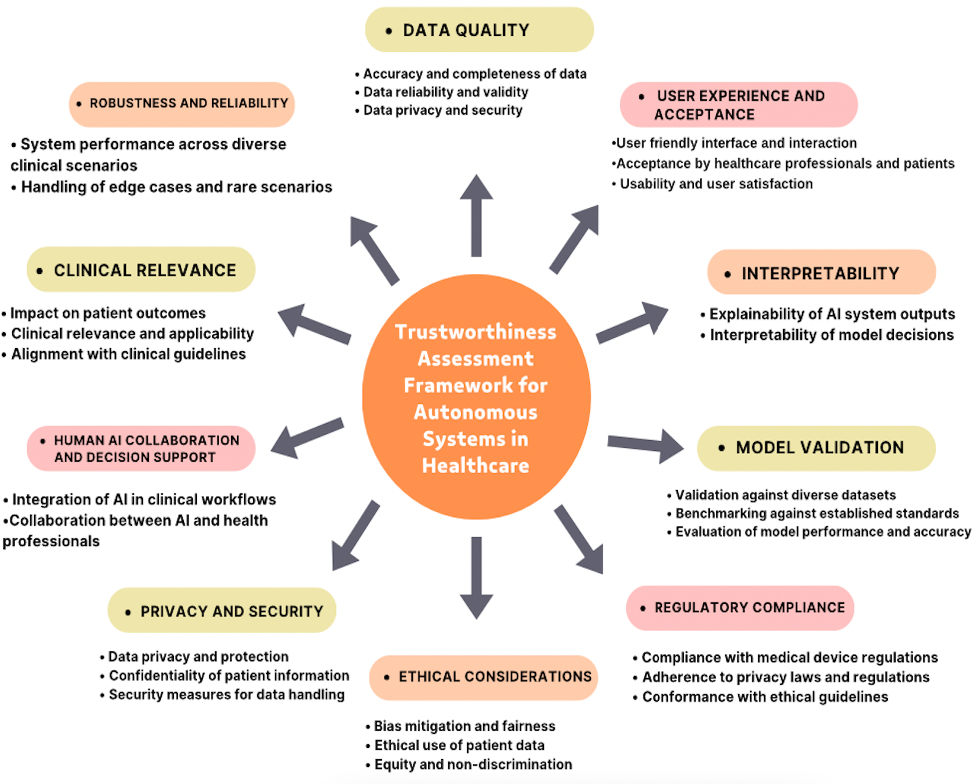

Supplement: Supplementary file 1 [file Image1.png]

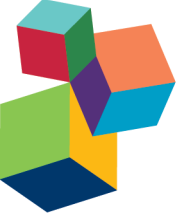

frontiers

Supplement: Supplementary file 2 [file Datasheet1.zip › logo1.pdf]

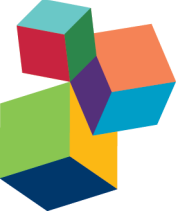

Supplement: Supplementary file 2 [file Datasheet1.zip › logo2.pdf]

A

frontiers  
FOR YOUNG MINDS

B

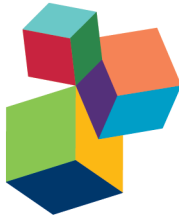

Supplement: Supplementary file 2 [file Datasheet1.zip › logos.pdf]
